# Clinical and bone-turnover outcomes associated with topical herbal plaster adjunctive therapy in older patients with myasthenia gravis and glucocorticoid-associated osteoporotic fragility fracture: a retrospective cohort study with exploratory peripheral-blood BMP-2/Smad/Runx2 analysis

**DOI:** 10.3389/fimmu.2026.1929809

**Published:** 2026-10-19

**Authors:** Qing Pan, Wei Wei, Chengjie Tang

**Affiliations:** 1 Department of Geriatric Orthopaedics, Sichuan Province Orthopedic Hospital, Chengdu, Sichuan, China; 2 Department of Anesthesiology, Affiliated Sport Hospital of CDSU, Chengdu, Sichuan, China

**Keywords:** bone turnover, fracture-site pain, glucocorticoid-associated osteoporosis, myasthenia gravis, osteoporotic fragility fracture, P1NP, Xiaotong plaster, β-CTx

## Abstract

**Background:**

Elderly patients with myasthenia gravis (MG) frequently require prolonged glucocorticoid therapy and may develop glucocorticoid-associated osteoporosis and fragility fracture. Post-fracture recovery in this population may be complicated by neuromuscular weakness, fall risk, impaired bone remodeling, fracture-site pain, and limited tolerance of systemic analgesic therapy. Xiaotong plaster is a topical external therapy used for musculoskeletal pain and swelling, but evidence for its adjunctive use after osteoporotic fracture in elderly patients with MG remains limited.

**Objective:**

This study evaluated the association between Xiaotong plaster adjunctive external treatment and 12-week fracture-site pain in elderly MG patients with glucocorticoid-associated osteoporotic fragility fracture. Bone-turnover and inflammatory outcomes were assessed as secondary exploratory endpoints, and BMP-2/Smad/Runx2-related changes were evaluated as hypothesis-generating mechanistic signals. These peripheral-blood measurements were treated as exploratory correlates and not as evidence that the topical plaster activated an osteogenic pathway at the fracture site.

**Methods:**

Consecutive patients aged 60 years or older with confirmed MG, systemic glucocorticoid exposure for at least 3 months, and radiologically confirmed osteoporotic fragility fracture were retrospectively screened at a tertiary medical center between January 2021 and December 2025. Patients receiving standard anti-osteoporosis treatment alone were compared with those receiving standard treatment plus Xiaotong plaster within 4 weeks after fracture. The primary outcome was fracture-site pain change from baseline to 12 weeks. Secondary and biomarker outcomes were considered exploratory. Propensity score weighting was used to reduce measured baseline imbalance, with doubly robust and sensitivity analyses used to examine the robustness of the primary association. Treatment allocation was observational and unblinded; no placebo plaster was used.

**Results:**

A total of 168 patients were included, including 86 receiving standard treatment alone and 82 receiving standard treatment plus Xiaotong plaster. The adjusted between-group difference in 12-week pain change was −10.2 mm on the 0–100 mm scale (95% CI, −13.8 to −6.6; P < 0.001). P1NP increased more in the adjunctive-treatment group (adjusted difference, 10.2 ng/mL; 95% CI, 6.8 to 13.6; P < 0.001), while β-CTX decreased modestly more (adjusted difference, −0.045 ng/mL; 95% CI, −0.087 to −0.003; P = 0.035). Six-month radiographic healing (P = 0.711), delayed union or nonunion (P = 0.363), 12-month new fragility fracture (P = 0.083), lumbar spine BMD change (P = 0.177), and MG exacerbation (P = 0.692) did not differ significantly after adjustment. In the exploratory subgroup, p-Smad1/5/8 (P = 0.001) and Runx2 (P = 0.002) increased more in the adjunctive-treatment group, whereas BMP-2 change was not significant (P = 0.449). The biomarker signal was therefore confined to downstream p-Smad1/5/8 and Runx2 rather than BMP-2 itself. One exposed patient permanently discontinued the plaster because of contact dermatitis.

**Conclusion:**

Xiaotong plaster adjunctive treatment was associated with greater short-term fracture-site pain reduction and exploratory changes in bone-turnover markers in elderly MG patients with glucocorticoid-associated osteoporotic fragility fracture. The study did not demonstrate improvement in fracture healing, BMD, or recurrent fracture, and the peripheral-blood BMP-2/Smad/Runx2 findings should be interpreted as hypothesis-generating rather than evidence of a causal osteogenic mechanism. Human transdermal pharmacokinetic exposure was not measured, so systemic pathway activation cannot be inferred from these data.

## 1 Introduction

Myasthenia gravis (MG) is an autoimmune neuromuscular junction disorder characterized by fluctuating skeletal muscle weakness and fatigability. A recent systematic review reported a mean MG prevalence of approximately 173 cases per million and a mean incidence of 15.7 cases per million person-years, with substantial geographic heterogeneity and increasing reported frequency over time (1). Older adults represent an increasingly important part of the MG population and may experience frailty, reduced mobility, respiratory vulnerability, and multimorbidity. Systemic glucocorticoids remain an important component of MG treatment when rapid disease control is required or when steroid-sparing therapy is insufficient, delayed, contraindicated, or unavailable (2).

Bone health is clinically important in elderly patients with MG. Population-based evidence has shown an increased risk of osteoporosis in MG, particularly among corticosteroid-treated patients (3). Fracture-risk estimates in MG have not been uniform across cohorts, indicating that glucocorticoid exposure, disease severity, reduced mobility, fall risk, vitamin D status, concomitant medications, and background osteoporosis care may contribute jointly to skeletal vulnerability (4, 5). Once a fragility fracture occurs, neuromuscular weakness and reduced mobility may complicate mobilization and rehabilitation, while frailty, respiratory vulnerability, renal or gastrointestinal comorbidity, dizziness, and polypharmacy can limit escalation of systemic analgesics (2, 6).

Glucocorticoid-associated osteoporosis is not fully captured by bone mineral density alone. Long-term glucocorticoid exposure suppresses osteoblast differentiation and function, affects osteocyte survival, alters bone remodeling balance, and increases fracture risk through quantitative and qualitative skeletal changes (7). Current guidance emphasizes early fracture-risk assessment, bone mineral density evaluation, calcium and vitamin D optimization, and appropriate anti-osteoporosis pharmacotherapy for patients at elevated risk (6). These systemic strategies are essential for fracture prevention and skeletal protection but are not designed primarily to provide local short-term analgesia after an established fracture.

Bone-turnover markers provide a practical way to describe early systemic bone remodeling before measurable bone mineral density change becomes apparent. Serum procollagen type I N-terminal propeptide (P1NP) reflects bone formation, whereas β-C-terminal telopeptide of type I collagen (β-CTX) reflects bone resorption; both are established reference markers for osteoporosis research and treatment monitoring (8). In glucocorticoid-exposed patients with fragility fracture, these markers may help characterize short-term remodeling responses, but they are systemic and should not be interpreted as direct measures of local fracture-site healing.

The BMP-2/Smad/Runx2 axis is biologically relevant to osteoblast differentiation and bone formation. BMP signaling can activate Smad-dependent pathways and interact with Runx2-mediated osteogenic transcription (9, 10). However, pathway markers measured in peripheral blood or archived peripheral blood mononuclear cells cannot substitute for local fracture-site tissue measurements. Accordingly, any pathway-related findings in this retrospective study were treated as exploratory biological signals rather than evidence of a treatment-mediated local mechanism.

Xiaotong plaster, also marketed as Qizheng Xiaotong plaster or CheeZheng pain-relieving plaster in the literature, is a topical traditional medicine preparation used for musculoskeletal pain and swelling. Existing clinical evidence is concentrated mainly in osteoarthritis and other musculoskeletal pain conditions rather than osteoporotic fracture in MG. A systematic review identified 22 randomized clinical trials involving 2,556 participants and reported potentially favorable pain and functional outcomes, but the review also noted inadequate reporting of randomization and an absence of placebo-controlled trials, limiting the certainty of efficacy estimates (11). Thus, previous evidence supports the plausibility of a local symptomatic role but does not establish an effect on osteoporosis, fracture healing, or immune regulation.

A clinically important evidence gap therefore remains. Elderly patients with MG and glucocorticoid-associated fragility fracture represent a narrow, high-risk population in whom local pain management may be useful, yet evidence specific to this population is lacking. In addition, it is uncertain whether any short-term symptomatic association is accompanied by measurable changes in systemic bone-turnover or inflammatory markers, and there is insufficient evidence to infer a local osteogenic mechanism from peripheral biomarkers. The primary objective of this retrospective cohort study was therefore to compare 12-week fracture-site pain change between standard anti-osteoporosis treatment alone and standard treatment plus Xiaotong plaster. Bone-turnover, inflammatory, longer-term skeletal, neurological, and safety outcomes were treated as secondary exploratory endpoints, and BMP-2/Smad/Runx2-related changes were examined in an availability-defined exploratory subgroup.

## 2 Materials and methods

### 2.1 Study design and data sources

This retrospective cohort study was conducted at Sichuan Province Orthopedic Hospital, a tertiary medical center, using routinely collected clinical data from the neurology inpatient system, orthopedic inpatient system, outpatient follow-up records, pharmacy prescription database, nursing medication records, laboratory information system, radiology archive, dual-energy X-ray absorptiometry reports, and electronic discharge summaries. Consecutive elderly patients with MG, systemic glucocorticoid exposure, and osteoporotic fragility fracture were screened between January 1, 2021, and December 31, 2025. The index date was defined as the date of radiologic confirmation of the qualifying fragility fracture. The study followed the STROBE statement and the RECORD extension for observational studies using routinely collected health data (12, 13).

Data sources, extraction hierarchy, and assessment windows are summarized in **Table 1**, and operational definitions and analytic handling are summarized in **Table 2**. For this retrospective analysis, eligibility criteria, exposure definitions, outcome definitions, and the core covariate set were formalized before comparative treatment-outcome models were fitted; this wording does not imply prospective registration or randomized allocation. Cohort construction is reported with the study results in **Figure 1**.

**Table 1:** Data sources, extraction hierarchy, and assessment windows.

Data domain	Primary source	Assessment window	Extraction hierarchy and adjudication	Main analysis use
Eligibility and demographics	Electronic medical record; discharge summary	Index admission	Age, sex, height, weight, admission diagnosis, fracture diagnosis, and discharge diagnosis were extracted from structured fields and verified against discharge summaries when inconsistent.	Cohort screening; baseline description
MG diagnosis	Neurology notes; antibody reports; electrophysiology reports	Before index fracture to 14 days after fracture	Specialist-confirmed MG diagnosis was prioritized. When diagnostic sources differed, neurology documentation was used as the reference source.	Eligibility confirmation
MG severity	Neurology notes; MG-ADL and QMG records	30 days before to 14 days after fracture	The score closest to the index fracture date was used. If inpatient and outpatient values were available on the same date, neurologist-recorded scores were prioritized.	Severity adjustment; subgroup analysis
Glucocorticoid exposure	Inpatient medication orders; outpatient prescriptions; pharmacy refill records	From MG diagnosis to index fracture; follow-up to 12 months	All systemic glucocorticoids were converted to prednisone-equivalent dose. Current dose, 3-month mean dose, cumulative dose, and exposure duration were extracted before outcome analysis.	Exposure characterization; confounding control
Standard anti-osteoporosis treatment	Pharmacy prescription database; discharge medication list; outpatient records	Index fracture to 12 months	Calcium, vitamin D, bisphosphonates, denosumab, teriparatide, and sequential therapy were extracted with start date, duration, and documented persistence.	Background treatment adjustment; sensitivity analysis
Xiaotong plaster exposure	Pharmacy dispensing records; inpatient medication orders; nursing application records; outpatient prescriptions	Index fracture to 12 weeks	Exposure required documented Xiaotong plaster use for at least 14 cumulative application days. Other topical analgesic patches or external herbal plasters during the same window were treated as treatment contamination in the primary comparison.	Main treatment exposure
Plaster adherence and interruption	Nursing records; outpatient follow-up notes; prescription refill records	Index fracture to 12 weeks	Adherence was calculated as documented application days divided by prescribed application days. Good adherence was defined as at least 80% completion. Interruption reason was extracted when available.	Exposure intensity; sensitivity analysis
Fracture characteristics	Radiology archive; orthopedic notes; operative records	Index admission and follow-up	Fracture site, vertebral level, laterality, displacement, surgical treatment, vertebral augmentation, and healing documentation were extracted. Ambiguous imaging findings were reviewed by two clinicians.	Fracture classification; outcome adjustment
Bone density and fracture risk	DXA report; osteoporosis clinic record; FRAX record	12 months before to 4 weeks after fracture	Lumbar spine, femoral neck, and total hip results were extracted. FRAX probability with femoral neck BMD was prioritized when available.	Baseline bone status; confounding control
Bone-turnover markers	Laboratory information system	Baseline, 12 weeks, 6 months, and 12 months	Baseline value was the result closest to the index fracture date within 14 days before to 7 days after fracture. Fasting morning values were prioritized for P1NP and β-CTX.	Bone metabolic profile and treatment response
Immune-inflammatory markers	Laboratory information system	Baseline and 12 weeks	CRP, IL-6, TNF-α, neutrophil count, lymphocyte count, and NLR were extracted. Extreme inflammatory values were checked against infection and perioperative records.	Inflammatory profile; confounding control
BMP-2/Smad/Runx2 subgroup	Archived serum or PBMC specimens; laboratory records	Baseline and 12 weeks	Only patients with valid paired specimens were included. Specimens with hemolysis, insufficient volume, incomplete pairing, repeated freeze–thaw cycles beyond the predefined quality-control limit, or failed internal control were excluded.	Exploratory biomarker analysis
Pain assessment	Inpatient notes; outpatient follow-up records; rehabilitation records	Baseline and 12 weeks	VAS was prioritized. If a 0–10 numeric rating scale was used, values were converted to a 0–100 mm scale.	Primary outcome
Radiographic healing	Radiology archive; orthopedic notes; operative records	Index fracture to 6 months	Site-specific healing status was extracted from radiology and orthopedic documentation. Unclear cases were reviewed by two clinicians.	Secondary outcome
New fragility fracture	Outpatient records; emergency records; readmission records; radiology archive	Index fracture to 12 months	New low-trauma fractures after the index date were counted as recurrent fragility fractures. Suspected events required radiologic confirmation.	Secondary time-to-event outcome
Safety events	Nursing notes; outpatient records; dermatology consultation; emergency and readmission records	Index fracture to 12 months	Plaster-related skin reactions and systemic adverse events were independently reviewed by two clinicians, with disagreement resolved by a senior clinician.	Safety analysis

**Table 2:** Operational measurement, reproducibility control, and analytic handling.

Construct	Operational measurement	Unit or coding	Reproducibility and quality-control rule	Handling in analysis
MG severity	MGFA class, MG-ADL score, and QMG score	MGFA class; score points	Scores closest to the index fracture date were used within the defined assessment window. Neurologist-recorded values were prioritized.	Baseline covariates; subgroup variables
Glucocorticoid exposure	Current prednisone-equivalent dose, 3-month mean dose, cumulative dose, and exposure duration	mg/day; g; months	Non-prednisone glucocorticoids were converted to prednisone-equivalent dose before analysis.	Core confounders
Standard anti-osteoporosis treatment	Calcium, vitamin D, bisphosphonate, denosumab, teriparatide, or sequential therapy	Treatment category; persistence status	Treatment start date, duration, and documented continuation were cross-checked across pharmacy and follow-up records.	Background treatment covariate; sensitivity analysis
Xiaotong plaster exposure	Documented cumulative application days within 12 weeks after fracture	Exposed/unexposed; days	Primary exposure threshold was at least 14 cumulative days. A stricter threshold of at least 28 cumulative days was used in sensitivity analysis.	Main exposure; sensitivity exposure
Xiaotong plaster adherence	Documented application days divided by prescribed application days	Percentage; ≥80% vs <80%	Application records, prescription records, and follow-up notes were cross-checked.	Adherence analysis; sensitivity analysis
Bone mineral density	DXA-derived lumbar spine, femoral neck, and total hip T-scores	T-score; g/cm²	Vertebrae affected by compression fracture, vertebroplasty, severe degeneration, metallic artifact, or structural abnormality were excluded from lumbar interpretation.	Baseline bone status; repeated BMD outcome
Vertebral fracture severity	Genant semiquantitative grading	Grade 0–3	Adequate lateral radiographs, sagittal CT reconstructions, or vertebral fracture assessment images were used. Uncertain cases were reviewed by two clinicians.	Fracture characterization
FRAX fracture risk	10-year major osteoporotic fracture and hip fracture probability	Percentage	FRAX with femoral neck BMD was prioritized when available. Glucocorticoid-dose adjustment was recorded when documented.	Baseline fracture-risk covariate
P1NP	Serum procollagen type I N-terminal propeptide	ng/mL	Fasting morning samples were prioritized. Original assay platform and collection date were retained.	Secondary bone-formation endpoint
β-CTX	Serum β-C-terminal telopeptide of type I collagen	ng/mL	Fasting morning samples were prioritized because of circadian variation.	Secondary bone-resorption endpoint
Osteocalcin	Serum osteocalcin	ng/mL	Values were extracted from the same laboratory system when available.	Secondary bone-turnover endpoint
25-hydroxyvitamin D	Serum 25-hydroxyvitamin D	ng/mL	Original laboratory result was retained and interpreted with supplementation status when available.	Baseline covariate
PTH	Serum or plasma parathyroid hormone	pg/mL	Values were interpreted with calcium, vitamin D, and renal function.	Baseline covariate
CRP	Serum C-reactive protein	mg/L	Extreme values were checked against infection records and perioperative status.	Immune-inflammatory marker; covariate
IL-6	Serum interleukin-6	pg/mL	Values below the lower detection limit were assigned half of the lower detection limit.	Secondary immune-inflammatory endpoint
TNF-α	Serum tumor necrosis factor-α	pg/mL	The same assay platform was preferred for paired baseline and 12-week values.	Secondary immune-inflammatory endpoint
NLR	Neutrophil count divided by lymphocyte count	Ratio	Counts from the same complete blood count report were used.	Baseline immune-inflammatory covariate
BMP-2	Serum BMP-2 concentration	pg/mL	Paired samples were measured in the same analytical batch whenever possible.	Exploratory biomarker endpoint
p-Smad1/5/8	Relative protein abundance in PBMC lysate	Relative expression	Expression was normalized to GAPDH or β-actin. Samples with failed internal control were excluded.	Exploratory biomarker endpoint
Runx2	Relative protein abundance in PBMC lysate	Relative expression	Expression was normalized to GAPDH or β-actin. Paired baseline and 12-week specimens from the same patient were analyzed together when possible.	Exploratory biomarker endpoint
Pain score	VAS or converted numeric rating scale	0–100 mm	VAS was prioritized. A 0–10 numeric rating scale was multiplied by 10 for harmonization.	Primary outcome
Radiographic healing	Site-specific radiologic and orthopedic documentation	Healed/not healed	Appendicular fractures required bridging callus or trabecular continuity. Vertebral fractures required stability without progressive collapse and clinical documentation of stabilization.	Secondary outcome
Delayed union or nonunion	Site-specific orthopedic or radiologic documentation	Yes/no	Events were recorded according to fracture site, treatment strategy, and 6-month follow-up documentation.	Secondary outcome
New fragility fracture	Radiologically confirmed low-trauma fracture after index date	Yes/no; time to event	Only fractures after the index date were counted. High-energy and pathological fractures were excluded.	Secondary time-to-event outcome
MG exacerbation	Worsening MG symptoms requiring treatment escalation or urgent medical evaluation	Yes/no	Events were verified using neurology notes, medication changes, emergency visits, admission records, IVIG use, or plasma exchange documentation.	Safety and neurological outcome
Plaster-related skin reaction	Local erythema, pruritus, blistering, contact dermatitis, ulceration, infection, or suspected allergy	Yes/no; severity; discontinuation	Relatedness was adjudicated by two reviewers. Events leading to interruption or discontinuation were recorded separately.	Safety outcome
Missing data	Missingness of candidate variables	Percentage missing	Variables with more than 20% missingness were excluded from the primary multivariable model unless part of the exploratory biomarker subgroup.	Imputation and sensitivity analysis

**Figure 1:**
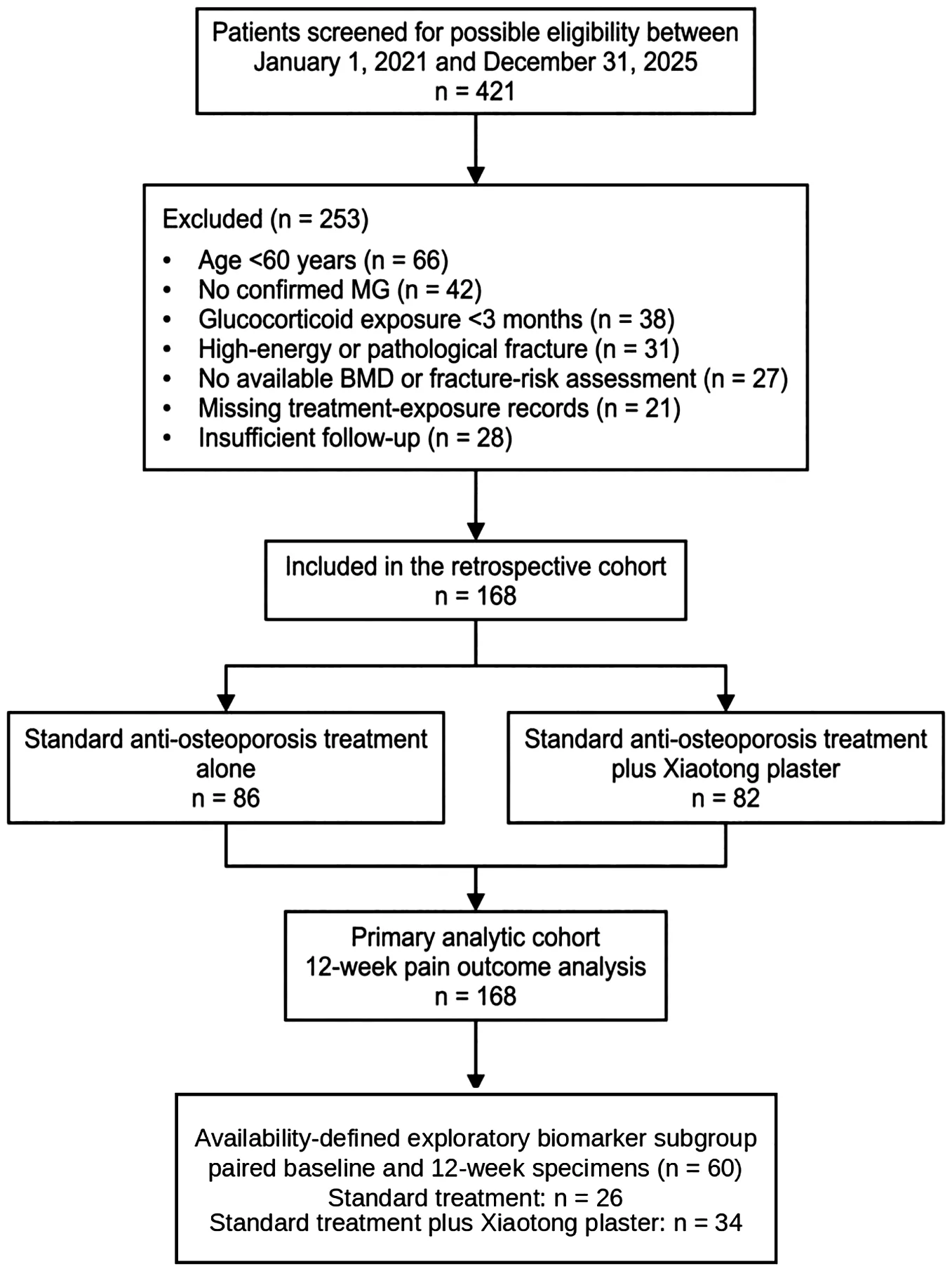
Cohort construction and analytic population. The flow diagram shows screened patients, exclusion steps, the final standard-treatment and Xiaotong plaster adjunctive-treatment groups, and the exploratory BMP-2/Smad/Runx2 subgroup defined by availability of valid paired baseline and 12-week specimens.

### 2.2 Participants

Patients were eligible if they were aged 60 years or older at the index fracture, had neurologist-confirmed MG before or at the index admission, had systemic glucocorticoid exposure for at least 3 months before fracture, had a radiologically confirmed low-trauma fragility fracture at a typical osteoporotic site, had baseline bone mineral density or fracture-risk assessment available, and had at least one post-fracture follow-up record within 12 months.

Major exclusion criteria were high-energy or pathological fracture, a major alternative metabolic or neoplastic bone disorder, end-stage kidney disease requiring dialysis, untreated endocrine conditions with a dominant effect on bone metabolism, glucocorticoid exposure primarily for a disease other than MG, missing index-fracture or treatment-exposure information, and insufficient post-fracture follow-up. For patients with more than one eligible fracture, the first qualifying fragility fracture was used as the index fracture.

For electronic case finding, the hospital diagnosis fields were screened using the ICD-10 category G70.0 (myasthenia gravis), supplemented by text-based MG diagnoses in neurology records. Both inpatient and outpatient diagnosis fields were included in the initial screen, and all candidate cases were subsequently confirmed from neurologist documentation and supporting antibody or electrophysiological evidence before inclusion.

Potentially eligible records were identified across the linked data sources listed in **Table 1**. MG diagnosis was then confirmed from neurology documentation and supporting antibody/electrophysiology records; glucocorticoid exposure was reconstructed from inpatient orders, outpatient prescriptions, discharge medication lists, and pharmacy refill records; qualifying fractures required radiologic confirmation. This multi-source confirmation was used because long-term MG care may span inpatient and outpatient settings.

### 2.3 MG diagnosis and neurological variables

MG diagnosis was confirmed by neurologists using compatible fluctuating fatigable weakness plus at least one supporting criterion, including positive acetylcholine receptor antibody or muscle-specific kinase antibody, abnormal repetitive nerve stimulation, abnormal single-fiber electromyography, documented response to acetylcholinesterase inhibitor therapy, or a previous specialist-confirmed MG diagnosis. MG subtype and severity were classified according to the Myasthenia Gravis Foundation of America clinical classification (14).

Baseline neurological variables included MG duration, ocular or generalized MG subtype, MGFA class, thymoma history, thymectomy history, acetylcholine receptor antibody status, muscle-specific kinase antibody status when available, pyridostigmine use, immunosuppressant use, intravenous immunoglobulin or plasma-exchange history, MG exacerbation within 6 months before fracture, MG Activities of Daily Living score, and Quantitative Myasthenia Gravis score (15, 16). Scores recorded within 30 days before to 14 days after the index fracture were accepted as baseline values. When multiple values were available, the value closest to the index fracture date was used.

### 2.4 Glucocorticoid exposure and standard anti-osteoporosis treatment

Systemic glucocorticoid exposure was extracted from inpatient medication orders, outpatient prescriptions, discharge medication lists, and pharmacy refill records. All systemic glucocorticoids were converted to prednisone-equivalent dose before analysis. Extracted variables included current daily prednisone-equivalent dose at the index fracture, mean daily dose during the preceding 3 months, cumulative prednisone-equivalent dose before fracture, duration of continuous glucocorticoid therapy, previous methylprednisolone pulse exposure, and glucocorticoid dose reduction during follow-up.

Standard anti-osteoporosis treatment was defined as physician-prescribed bone-protective therapy after fracture, including calcium carbonate or calcium citrate, vitamin D or active vitamin D, oral bisphosphonate, intravenous zoledronic acid, denosumab, teriparatide, or sequential therapy selected according to renal function, gastrointestinal tolerance, fracture risk, prior anti-osteoporosis treatment, and contraindications. Treatment start date, treatment category, documented persistence, and treatment interruption were extracted from pharmacy and follow-up records. The definition of clinically relevant glucocorticoid exposure and the need for fracture-risk assessment followed current glucocorticoid-induced osteoporosis guidance (17).

### 2.5 Xiaotong plaster adjunctive external treatment

The adjunctive external-treatment group included patients who received standard anti-osteoporosis treatment plus Xiaotong plaster within 4 weeks after the index fracture. Xiaotong plaster, also marketed as Qizheng Xiaotong plaster, is a nationally approved external traditional medicine plaster with the approval number Z54020113. According to the product information, each package contains a medicated core bag and wetting agent; the specification is 1.2 g per medicated core bag and 2.5 mL per wetting-agent bag. The product is externally applied by spreading the wetting agent evenly onto the medicated pad and then applying the moistened pad to the painful site or a clinically selected acupoint region. Each application lasts 24 h. The product information describes its function as promoting blood circulation, relieving swelling, and reducing pain, and lists indications including acute and chronic sprain, contusion-related pain, osteoproliferative pain, rheumatic or rheumatoid pain, stiff neck, shoulder periarthritis, lumbar muscle strain, and old injury-related pain (18). Published clinical descriptions identify Lamiophlomis rotata (Duyiwei) and Curcuma longa (Jianghuang) as principal herbal components of the marketed plaster, with additional botanical constituents described in some formulations; accordingly, Xiaotong plaster is a multicomponent topical preparation rather than a single-active drug (11, 18). A product-specific preclinical study showed that luteolin from Xiaotong plaster crossed rat skin and was detectable in subcutaneous muscle and bone tissue, but this animal study does not establish human systemic exposure or pharmacokinetics (19).

In this study, Xiaotong plaster was not defined as a systemic anti-osteoporosis drug and was not used as a substitute for calcium, vitamin D, bisphosphonates, denosumab, teriparatide, or other standard anti-osteoporosis therapy. It was evaluated as an adjunctive external treatment for fracture-site pain and local functional recovery after osteoporotic fragility fracture. This positioning was consistent with the clinical use of topical traditional medicine interventions as supportive local treatment for pain, swelling, and functional symptoms after osteoporotic fracture (20).

Xiaotong plaster exposure was identified from pharmacy dispensing records, inpatient medication orders, nursing application records, outpatient prescriptions, follow-up documentation, and discharge medication lists. The primary exposure definition required at least 14 documented cumulative application days within the first 12 weeks after the index fracture; a stricter threshold of at least 28 cumulative days was evaluated in sensitivity analysis. The comparator group included patients receiving standard anti-osteoporosis treatment without documented Xiaotong plaster exposure during the same 12-week window.

For vertebral compression fracture, the plaster was applied to intact paraspinal skin corresponding to the painful vertebral region. For hip, distal radius, proximal humerus, pelvis, or rib fracture, the plaster was applied to intact skin around the painful region rather than directly over an open wound, surgical incision, fixation site, ulcerated skin, or infected skin. In surgically treated fractures, application was started only after the incision was dry, closed, and clinically stable. Patients with known plaster allergy, active dermatitis at the intended application site, open traumatic wound, or documented intolerance to previous Xiaotong plaster use were not classified as exposed. Use of other topical analgesic patches, non-steroidal anti-inflammatory patches, opioid transdermal patches, or other topical herbal plasters during the same exposure window was recorded as treatment contamination. Patients whose Xiaotong plaster exposure could not be distinguished from other topical treatments were excluded from the primary exposure definition.

Documented application days were counted from the available treatment records across inpatient and outpatient care. Prescribed application days were obtained from the corresponding medication orders or prescriptions, and adherence was calculated as documented application days divided by prescribed application days × 100%. Good adherence was defined as at least 80% completion. Nursing application records, outpatient follow-up notes, prescription records, and refill information were cross-checked where available. Start date, cumulative application days, application site, interruption, reason for discontinuation, adherence category, and plaster-related skin reaction were extracted. Because these measures depended on routine documentation, potential exposure misclassification was addressed in sensitivity analyses using stricter exposure and adherence definitions.

### 2.6 Fracture confirmation, bone density, and fracture-risk assessment

A fragility fracture was defined as a fracture occurring after a fall from standing height or less, minimal trauma, or no identifiable trauma at a skeletal site typical of osteoporosis (21). Fracture diagnosis was confirmed using radiography, computed tomography, or magnetic resonance imaging. Fracture site, vertebral level when applicable, laterality, displacement, fracture-management strategy, surgical procedure, vertebral augmentation, and follow-up imaging status were extracted from orthopedic and radiology records.

Bone mineral density was measured by dual-energy X-ray absorptiometry. Lumbar spine, femoral neck, and total hip T-scores were extracted. Vertebrae affected by compression fracture, vertebroplasty, severe degenerative change, metallic artifact, or other structural abnormality were excluded from lumbar-spine interpretation according to adult densitometry reporting principles (22). Osteoporosis was defined as a T-score of −2.5 or lower at the lumbar spine, femoral neck, or total hip, or the presence of a clinically recognized fragility fracture.

The 10-year probability of major osteoporotic fracture and hip fracture was calculated using FRAX with femoral neck bone mineral density when available; glucocorticoid-dose adjustment was recorded when documented in the clinical file (23). Vertebral compression fractures were graded using the Genant semiquantitative method when adequate lateral spine radiographs, sagittal CT reconstructions, or vertebral fracture assessment images were available (24).

### 2.7 Laboratory, bone-turnover, and inflammatory variables

Baseline laboratory values were defined as results closest to the index fracture date within 14 days before to 7 days after fracture. Follow-up values were extracted at 12 weeks, 6 months, and 12 months when available. Fasting morning samples were prioritized for bone-turnover markers.

Routine laboratory variables included serum calcium, phosphorus, albumin, alkaline phosphatase, creatinine, estimated glomerular filtration rate, 25-hydroxyvitamin D, parathyroid hormone, hemoglobin, fasting glucose, C-reactive protein, neutrophil count, lymphocyte count, and neutrophil-to-lymphocyte ratio. Bone-turnover variables included procollagen type I N-terminal propeptide, β-C-terminal telopeptide of type I collagen, and osteocalcin. Immune-inflammatory variables included interleukin-6, tumor necrosis factor-α, C-reactive protein, and neutrophil-to-lymphocyte ratio.

### 2.8 Exploratory peripheral-blood BMP-2/Smad/Runx2 biomarker subgroup

The BMP-2/Smad/Runx2 analysis was an exploratory subgroup analysis restricted to all patients for whom valid paired baseline and 12-week archived serum or peripheral blood mononuclear cell specimens were available and passed pre-analytical quality control. The subgroup size was therefore availability-defined rather than determined by a separate power calculation. Serum BMP-2 was measured by sandwich enzyme-linked immunosorbent assay. Phosphorylated Smad1/5/8 and Runx2 expression were measured in peripheral blood mononuclear cell lysates using capillary western blotting or conventional western blotting and normalized to GAPDH or β-actin. Results were expressed as relative protein abundance. These measurements were used only to explore pathway-associated signals and were not interpreted as direct evidence of local fracture-site biology. No absorption, plasma pharmacokinetic, or fracture-site tissue measurements were available to connect topical exposure with these peripheral markers.

Paired samples from the same patient were measured in the same analytical batch whenever possible. Laboratory staff were blinded to treatment group and clinical outcome status. Samples were excluded from mechanistic analysis if they had visible hemolysis, insufficient volume, incomplete time-pairing, more than two freeze–thaw cycles, or failed internal control.

### 2.9 Outcomes

The primary clinical outcome was change in fracture-site pain from baseline to 12 weeks. Pain was extracted using a 0–100 mm visual analogue scale; when a 0–10 numeric rating scale was recorded, the value was multiplied by 10 for harmonization. Twelve weeks was selected as an early recovery endpoint because pain from acute or subacute osteoporotic fracture, particularly vertebral fracture, commonly evolves over an approximately 8–12-week period during conservative recovery (25). Because the present cohort included multiple fragility fracture sites, the applicability of a uniform 12-week pain endpoint across heterogeneous fracture types is not fully established; the 12-week assessment should therefore be interpreted as a pragmatic early-recovery endpoint rather than a universally validated pain time point for all fracture types.

Secondary outcomes included 12-week changes in P1NP, β-CTX, osteocalcin, interleukin-6, tumor necrosis factor-α, and C-reactive protein; 6-month radiographic fracture healing; delayed union or nonunion at 6 months; 12-month change in lumbar spine, femoral neck, and total hip bone mineral density; new fragility fracture within 12 months; MG exacerbation within 12 months; systemic anti-osteoporosis treatment persistence; all-cause readmission; and plaster-related skin reaction. These endpoints were included to describe complementary short-term metabolic, longer-term skeletal, neurological, and safety outcomes; the fixed retrospective cohort of 168 patients was not designed or separately powered to provide confirmatory inference for each secondary endpoint. Radiographic healing definitions remained site-specific as described below.

MG exacerbation was defined as worsening ocular, bulbar, limb, respiratory, or generalized MG symptoms requiring treatment escalation, urgent neurology evaluation, emergency care, hospitalization, intravenous immunoglobulin, plasma exchange, or clinically documented rescue therapy. Plaster-related skin reaction was defined as local erythema, pruritus, blistering, contact dermatitis, ulceration, infection, or suspected allergy occurring at or near the application site during the exposure window or follow-up.

### 2.10 Confounding control

Potential confounders were selected before outcome analysis based on clinical relevance and the expected relationship with both plaster prescription and outcome. The core adjustment set included age, sex, body mass index, fracture site, fracture-management strategy, baseline pain score, baseline systemic analgesic use, MGFA class, MG-ADL score, QMG score, baseline lumbar spine T-score, femoral neck T-score, baseline P1NP, baseline β-CTX, baseline 25-hydroxyvitamin D, baseline C-reactive protein, current prednisone-equivalent dose, cumulative prednisone-equivalent dose, glucocorticoid duration, use of bisphosphonate, use of denosumab or teriparatide, renal function, diabetes, and prior fragility fracture.

Because Xiaotong plaster exposure was not randomly assigned, propensity score weighting was used as the main method to reduce measured baseline imbalance (26). The propensity score was estimated using logistic regression with Xiaotong plaster exposure as the dependent variable and the clinically selected baseline covariates as independent variables. Stabilized inverse probability of treatment weights were calculated. Balance was assessed using standardized mean differences (SMDs), with an absolute SMD below 0.10 used as the target threshold. Covariate balance before and after weighting was displayed in a Love plot.

If selected covariates remained above the target balance threshold after weighting, they were retained in a doubly robust weighted outcome model to reduce the influence of residual measured imbalance. Extreme weights were truncated at the 1st and 99th percentiles in sensitivity analysis.

### 2.11 Statistical analysis

Continuous variables were summarized as mean with standard deviation or median with interquartile range according to distribution. Categorical variables were summarized as number and percentage. Baseline characteristics were described before and after propensity score weighting, and between-group baseline differences were interpreted using standardized mean differences rather than significance testing.

The primary analysis estimated the association between Xiaotong plaster adjunctive treatment and 12-week fracture-site pain change using inverse probability of treatment-weighted linear regression with robust standard errors. Secondary continuous outcomes, including changes in P1NP, β-CTX, osteocalcin, inflammatory markers, and bone mineral density, were analyzed using weighted linear regression. Binary outcomes, including 6-month radiographic healing, delayed union or nonunion, MG exacerbation, all-cause readmission, and plaster-related skin reaction, were analyzed using weighted logistic regression when model convergence was adequate. New fragility fracture within 12 months was analyzed using weighted Cox proportional hazards regression and reported as hazard ratio with 95% confidence interval. The proportional hazards assumption was assessed using Schoenfeld residuals.

For the primary outcome, a doubly robust weighted model was additionally performed when clinically important baseline covariates remained above the target standardized mean difference threshold after weighting. Exploratory biomarker endpoints were analyzed only in patients with valid paired baseline and 12-week specimens. Correlations between pathway-marker changes and bone-turnover or clinical changes were evaluated using Spearman correlation coefficients. Because the study had one primary clinical outcome and a broad set of secondary, subgroup, sensitivity, and biomarker analyses, no formal multiplicity adjustment was applied to those exploratory analyses; their nominal P values and confidence intervals were used descriptively and were not interpreted as confirmatory evidence. No formal mediation model or causal pathway model linking plaster exposure, peripheral biomarkers, and pain or healing was fitted.

Missingness was summarized for all candidate variables. Variables with more than 20% missingness were not entered into the primary multivariable model unless they belonged to the exploratory mechanistic subgroup. For variables with 20% or less missingness, multiple imputation by chained equations was used under a missing-at-random assumption (27), with complete-case analysis as a sensitivity analysis. Two-sided P values below 0.05 were used for the primary outcome. Secondary, subgroup, sensitivity, and biomarker P values were explicitly interpreted as exploratory in view of multiple comparisons and limited power for many secondary outcomes. Analyses were performed using R software with reproducible scripts.

### 2.12 Sensitivity and subgroup analyses

Sensitivity analyses were performed to examine the robustness of the primary 12-week pain outcome. These analyses included complete-case analysis, propensity score matching using a 1:1 nearest-neighbor algorithm with a caliper of 0.2 standard deviations of the logit of the propensity score, reclassification of Xiaotong plaster exposure using a stricter threshold of at least 28 cumulative application days, restriction to patients with at least 80% documented Xiaotong plaster adherence, restriction to patients with at least 80% documented persistence with standard anti-osteoporosis therapy, exclusion of patients receiving teriparatide, exclusion of patients undergoing vertebral augmentation, truncation of extreme inverse probability weights at the 1st and 99th percentiles, and treatment of cumulative Xiaotong plaster application days as a continuous exposure.

Exploratory subgroup analyses for the primary 12-week pain outcome were performed by age group, sex, vertebral versus non-vertebral fracture, baseline prednisone-equivalent dose, baseline osteoporosis severity, fracture-management strategy, and systemic anti-osteoporosis treatment type. Age was categorized as younger than 75 years or 75 years or older. Baseline prednisone-equivalent dose was categorized as less than 10 mg/day or 10 mg/day or higher. Baseline osteoporosis severity was categorized using lumbar spine T-score greater than −2.5 or −2.5 or lower. Interaction terms were reported as exploratory and were not used for confirmatory inference.

### 2.13 Safety assessment

Safety outcomes were extracted from inpatient notes, outpatient follow-up records, nursing records, dermatology consultations, emergency visits, and readmission records. Plaster-related adverse events included local erythema, pruritus, blistering, contact dermatitis, skin ulceration, local infection, treatment interruption due to skin reaction, and documented allergy. Systemic safety outcomes included MG exacerbation, myasthenic crisis, infection requiring antibiotic treatment, hypercalcemia, renal-function deterioration, gastrointestinal intolerance related to systemic anti-osteoporosis treatment, and all-cause readmission within 12 months.

Two reviewers independently adjudicated adverse-event relatedness and severity, and disagreement was resolved by a senior clinician. Plaster-related skin reactions were described by event type, severity, need for treatment interruption, and resolution status. MG exacerbation and myasthenic crisis were verified using neurology documentation, treatment escalation records, rescue therapy, emergency visits, or hospitalization records.

## 3 Results

### 3.1 Cohort construction and baseline characteristics

A total of 421 patients with records suggestive of MG, systemic glucocorticoid exposure, and fracture were screened between January 1, 2021, and December 31, 2025. After excluding patients younger than 60 years (n = 66), those without confirmed MG (n = 42), those with systemic glucocorticoid exposure shorter than 3 months (n = 38), those with high-energy or pathological fracture (n = 31), those without available BMD or fracture-risk assessment (n = 27), those with missing treatment-exposure records (n = 21), and those with insufficient follow-up (n = 28), 168 patients were included in the final cohort. Among them, 86 received standard anti-osteoporosis treatment alone and 82 received standard anti-osteoporosis treatment plus Xiaotong plaster. The exploratory BMP-2/Smad/Runx2 subgroup comprised all 60 patients with valid paired baseline and 12-week specimens, including 26 in the standard-treatment group and 34 in the adjunctive-treatment group (**Figure 1**).

The mean age was 73.0 ± 6.9 years in the standard-treatment group and 74.3 ± 7.4 years in the adjunctive-treatment group. Female patients accounted for 69.8% and 73.2% of the two groups, respectively. Baseline characteristics of the study cohort before propensity score weighting are summarized in **Table 3**. Before weighting, clinically relevant imbalance was present in several covariates, including lumbar spine T-score, baseline P1NP, baseline β-CTX, teriparatide use, vertebral compression fracture distribution, baseline pain score, and distal radius fracture distribution. Propensity score weighting improved covariate balance overall. Residual imbalance above the prespecified target of 0.10 remained for teriparatide use (weighted SMD, 0.128) and vertebral compression fracture distribution (weighted SMD, 0.112); these variables were therefore retained in the doubly robust outcome model. The full balance pattern for the key imbalanced covariates is displayed in **Figure 2**.

**Figure 2:**
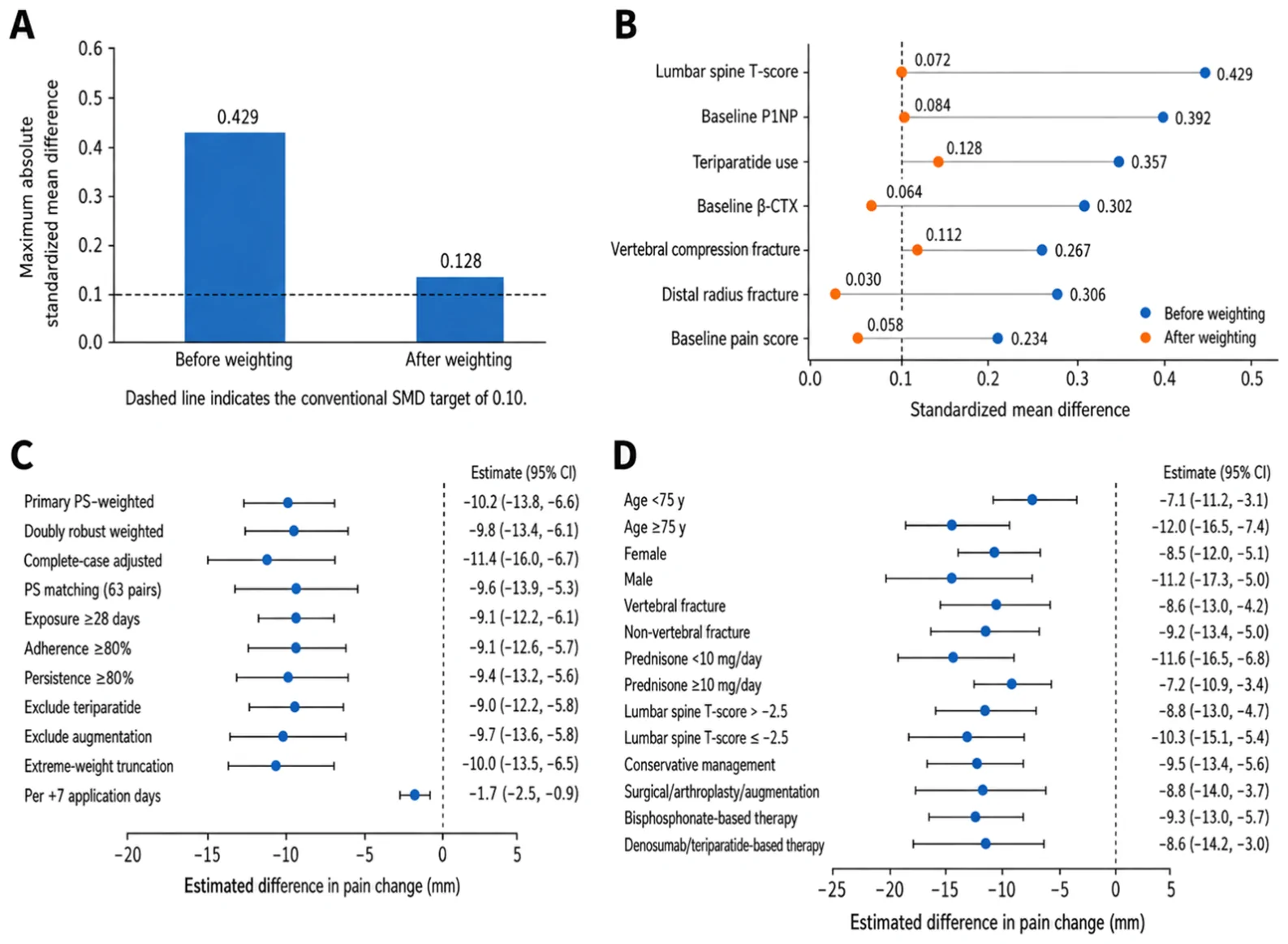
Propensity score balance and robustness analyses. **(A)** Maximum absolute standardized mean difference before and after propensity score weighting. **(B)** Love plot showing standardized mean differences for key imbalanced covariates before and after weighting. **(C)** Forest plot of sensitivity analyses for 12-week pain reduction. **(D)** Forest plot of exploratory subgroup analyses for 12-week pain reduction.

**Table 3:** Baseline characteristics of the study cohort before propensity score weighting.

Variable	Standard treatment only *n* = 86	Standard treatment plus Xiaotong plaster *n* = 82
Age, years	73.0 ± 6.9	74.3 ± 7.4
Female sex	60 (69.8%)	60 (73.2%)
BMI, kg/m²	23.1 ± 3.1	22.5 ± 3.2
Generalized MG	69 (80.2%)	68 (82.9%)
MG duration, years	8.5 ± 4.8	8.0 ± 4.6
MGFA class II	38 (44.2%)	31 (37.8%)
MGFA class III	35 (40.7%)	38 (46.3%)
MGFA class IV	13 (15.1%)	13 (15.9%)
MG-ADL score	7.5 ± 2.7	7.5 ± 3.3
QMG score	12.8 ± 4.8	13.7 ± 4.6
Acetylcholine receptor antibody positive	72 (83.7%)	68 (82.9%)
Muscle-specific kinase antibody positive	4 (4.7%)	3 (3.7%)
Pyridostigmine use	79 (91.9%)	74 (90.2%)
Other immunosuppressant use	54 (62.8%)	51 (62.2%)
MG exacerbation within 6 months before fracture	11 (12.8%)	13 (15.9%)
Current prednisone-equivalent dose, mg/day	10.9 ± 5.6	11.6 ± 5.6
Mean prednisone-equivalent dose during preceding 3 months, mg/day	12.4 ± 6.0	13.0 ± 6.3
Cumulative prednisone-equivalent dose, g	16.0 ± 4.5	17.2 ± 4.5
Glucocorticoid duration, months	91.3 ± 36.1	93.0 ± 33.5
Lumbar spine T-score	-2.30 ± 0.54	-2.54 ± 0.60
Femoral neck T-score	-2.15 ± 0.64	-2.26 ± 0.62
Total hip T-score	-2.03 ± 0.58	-2.12 ± 0.60
FRAX major osteoporotic fracture risk, %	22.4 ± 6.9	23.6 ± 6.8
FRAX hip fracture risk, %	10.1 ± 4.9	11.0 ± 4.9
Baseline fracture-site pain score, 0–100 mm	62.7 ± 14.3	65.8 ± 12.2
Baseline systemic analgesic use	43 (50.0%)	46 (56.1%)
Vertebral compression fracture	40 (46.5%)	49 (59.8%)
Hip fracture	15 (17.4%)	16 (19.5%)
Distal radius fracture	23 (26.7%)	10 (12.2%)
Proximal humerus fracture	8 (9.3%)	7 (8.5%)
Conservative fracture management	55 (64.0%)	51 (62.2%)
Surgical fixation or arthroplasty	19 (22.1%)	17 (20.7%)
Vertebral augmentation	12 (14.0%)	14 (17.1%)
Calcium/vitamin D only	11 (12.8%)	6 (7.3%)
Calcium/vitamin D plus bisphosphonate	53 (61.6%)	48 (58.5%)
Calcium/vitamin D plus denosumab	16 (18.6%)	15 (18.3%)
Calcium/vitamin D plus teriparatide	6 (7.0%)	13 (15.9%)
Documented persistence with standard anti-osteoporosis treatment ≥80%	65 (75.6%)	67 (81.7%)
Baseline P1NP, ng/mL	34.1 ± 12.1	29.7 ± 10.3
Baseline β-CTX, ng/mL	0.52 ± 0.19	0.46 ± 0.20
Baseline osteocalcin, ng/mL	14.9 ± 5.8	13.6 ± 5.5
Baseline 25-hydroxyvitamin D, ng/mL	18.7 ± 6.8	17.9 ± 7.0
Baseline CRP, mg/L	6.9 ± 6.4	7.0 ± 5.4
Baseline IL-6, pg/mL	8.8 ± 5.9	9.7 ± 7.2
Baseline TNF-α, pg/mL	12.1 ± 5.3	12.8 ± 5.7
Baseline NLR	2.74 ± 1.22	2.86 ± 1.31
Estimated glomerular filtration rate, mL/min/1.73 m²	73.5 ± 18.9	70.9 ± 20.2
Diabetes	19 (22.1%)	22 (26.8%)
Prior fragility fracture	26 (30.2%)	29 (35.4%)
BMP-2/Smad/Runx2 subgroup available	26 (30.2%)	34 (41.5%)

Values are presented as mean ± standard deviation or n (%). MG, myasthenia gravis; MGFA, Myasthenia Gravis Foundation of America; MG-ADL, Myasthenia Gravis Activities of Daily Living; QMG, Quantitative Myasthenia Gravis; FRAX, fracture risk assessment tool; P1NP, procollagen type I N-terminal propeptide; β-CTX, β-C-terminal telopeptide of type I collagen; CRP, C-reactive protein; IL-6, interleukin-6; TNF-α, tumor necrosis factor-α; NLR, neutrophil-to-lymphocyte ratio. **Table 3** presents unweighted descriptive values; standardized mean differences before and after propensity score weighting are summarized separately in **Figure 2**.

### 3.2 Baseline bone metabolic, inflammatory, and fracture profile

Baseline lumbar spine, femoral neck, and total hip T-scores were generally within the osteoporotic or low-bone-mass range in both groups, with a lower lumbar spine T-score in the adjunctive-treatment group before weighting (**Figure 3A**). Bone-turnover markers showed wide inter-individual dispersion (**Figure 3B**), while CRP, IL-6, and TNF-a showed scattered high values without a clear baseline separation between treatment groups. Because the inflammatory markers did not define a distinct treatment-group phenotype, these data are described as baseline bone metabolic and inflammatory characteristics rather than as evidence of a specific immune–bone metabolic state. At the treatment-group summary level, the adjunctive-treatment group had both a higher mean cumulative prednisone-equivalent dose and a lower mean lumbar spine T-score; because **Figure 3C** contains only two group-level summary points, no patient-level dose–BMD association is inferred from this panel. The baseline fracture-site distribution by treatment group is shown in **Figure 3D**.

**Figure 3:**
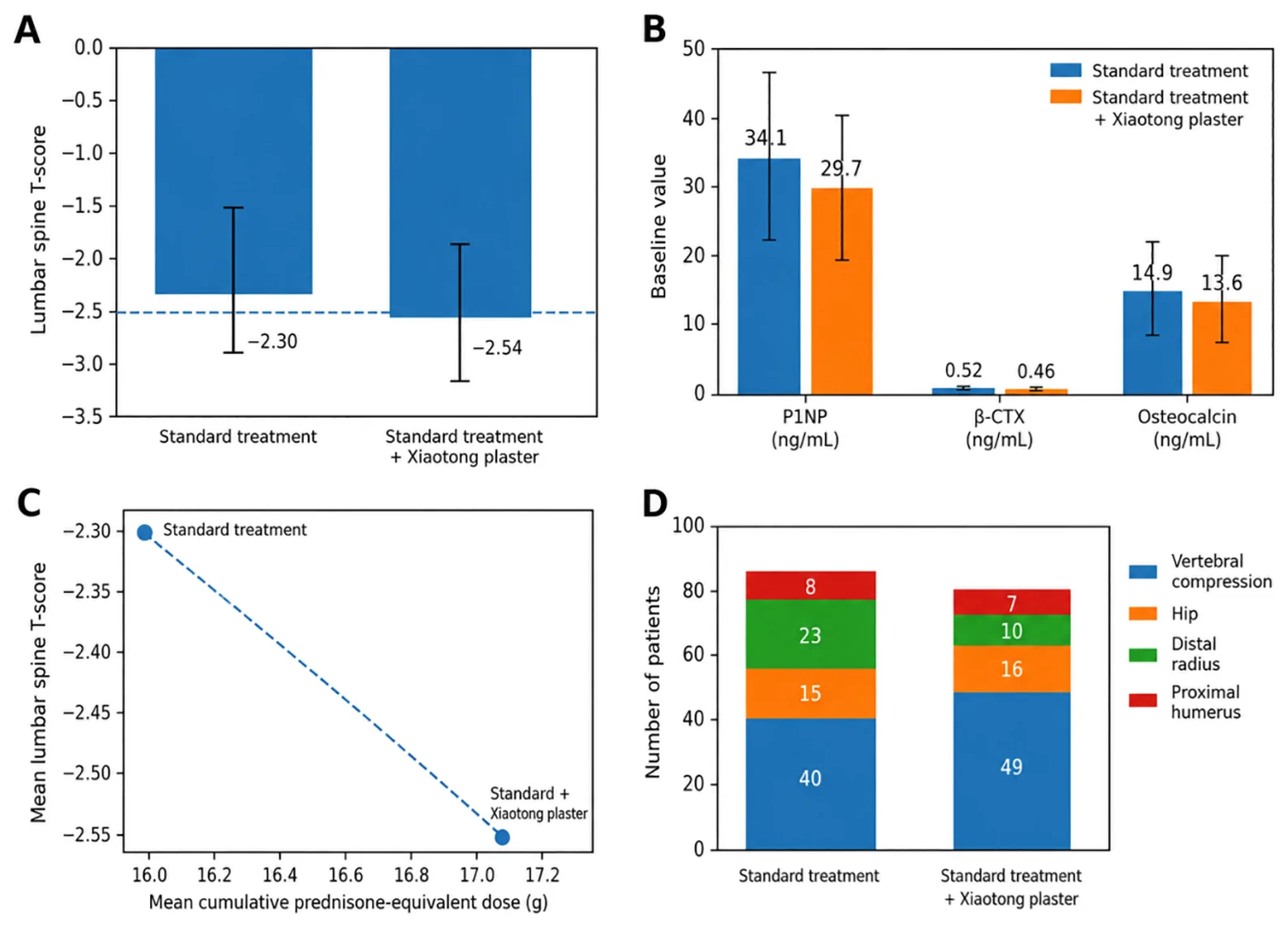
Baseline bone metabolic and fracture profile. **(A)** Group-level summary of lumbar spine T-score by treatment group. **(B)** Group-level summaries of baseline P1NP, β-CTX, and osteocalcin. **(C)** Descriptive group-level plot of mean cumulative prednisone-equivalent dose against mean lumbar spine T-score; because only two treatment-group summary points are shown, this panel is not interpreted as a patient-level correlation or smoothed dose–response relationship. **(D)** Stacked bar chart showing fracture-site distribution by treatment group.

### 3.3 Primary outcome and 12-week bone-turnover and inflammatory response

Fracture-site pain decreased in both groups by week 12, with a larger reduction in the Xiaotong plaster adjunctive-treatment group. Mean pain score decreased from 62.7 ± 14.3 to 46.5 ± 18.5 mm in the standard-treatment group and from 65.8 ± 12.2 to 40.4 ± 15.5 mm in the adjunctive-treatment group. After propensity score weighting, adjunctive Xiaotong plaster treatment was associated with an additional pain reduction of −10.2 mm at 12 weeks compared with standard treatment alone (95% CI, −13.8 to −6.6; P < 0.001).

P1NP increased more in the adjunctive-treatment group than in the standard-treatment group. The adjusted between-group difference in 12-week P1NP change was 10.2 ng/mL (95% CI, 6.8 to 13.6; P < 0.001). β-CTX decreased in both groups, with an adjusted between-group difference of −0.045 ng/mL (95% CI, −0.087 to −0.003; P = 0.035). Osteocalcin also showed a greater 12-week increase in the adjunctive-treatment group, whereas CRP, IL-6, and TNF-α changes were not clearly different between groups. These 12-week clinical, bone-turnover, and inflammatory changes are shown in **Figure 4** and summarized in **Table 4**.

**Figure 4:**
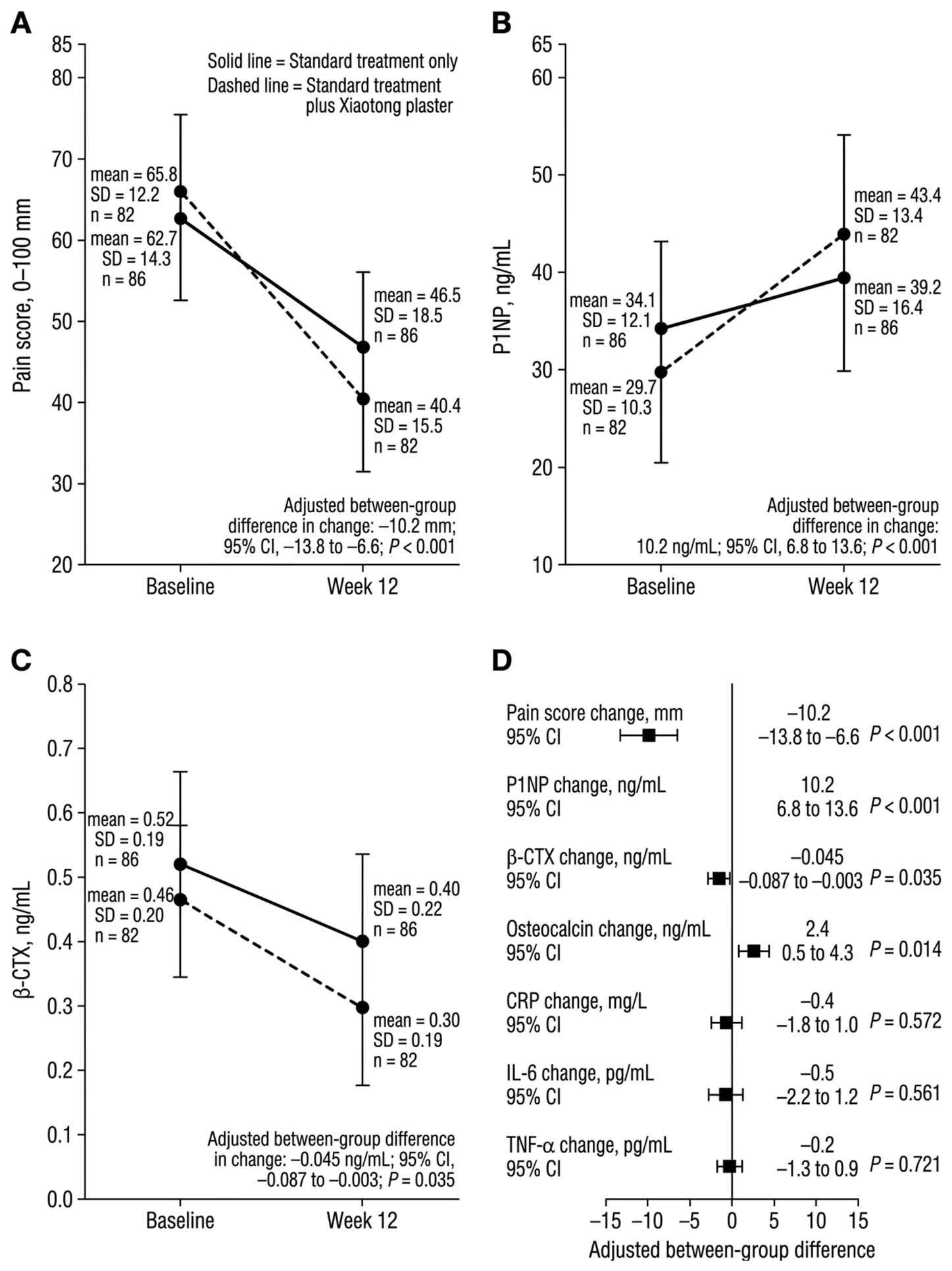
Twelve-week clinical, bone-turnover, and inflammatory response. **(A)** Group mean pain scores with variability at baseline and week 12. **(B)** Group mean P1NP values with variability at baseline and week 12. **(C)** Group mean β-CTX values with variability at baseline and week 12. **(D)** Forest plot of adjusted associations between Xiaotong plaster adjunctive treatment and 12-week changes in pain score, P1NP, β-CTX, osteocalcin, CRP, IL-6, and TNF-α.

**Table 4:** Twelve-week clinical, bone-turnover, and inflammatory outcomes.

Outcome	Standard treatment only	Standard treatment plus Xiaotong plaster	Adjusted treatment effect
Baseline pain score, 0–100 mm	62.7 ± 14.3	65.8 ± 12.2	—
Week-12 pain score, 0–100 mm	46.5 ± 18.5	40.4 ± 15.5	—
Pain change from baseline to week 12, mm	−16.2 ± 10.5	−25.4 ± 9.5	−10.2 mm; 95% CI, −13.8 to −6.6; P < 0.001
Baseline P1NP, ng/mL	34.1 ± 12.1	29.7 ± 10.3	—
Week-12 P1NP, ng/mL	39.2 ± 16.4	43.4 ± 13.4	—
P1NP change from baseline to week 12, ng/mL	5.7 ± 10.5	14.3 ± 8.9	10.2 ng/mL; 95% CI, 6.8 to 13.6; P < 0.001
Baseline β-CTX, ng/mL	0.52 ± 0.19	0.46 ± 0.20	—
Week-12 β-CTX, ng/mL	0.40 ± 0.22	0.30 ± 0.19	—
β-CTX change from baseline to week 12, ng/mL	−0.124 ± 0.106	−0.178 ± 0.100	−0.045 ng/mL; 95% CI, −0.087 to −0.003; P = 0.035
Baseline osteocalcin, ng/mL	14.9 ± 5.8	13.6 ± 5.5	—
Week-12 osteocalcin, ng/mL	16.2 ± 6.4	17.5 ± 6.1	—
Osteocalcin change from baseline to week 12, ng/mL	1.3 ± 5.2	3.9 ± 5.5	2.4 ng/mL; 95% CI, 0.5 to 4.3; P = 0.014
Baseline CRP, mg/L	6.9 ± 6.4	7.0 ± 5.4	—
Week-12 CRP, mg/L	5.8 ± 5.2	5.5 ± 4.9	—
CRP change from baseline to week 12, mg/L	−1.0 ± 4.2	−1.5 ± 4.5	−0.4 mg/L; 95% CI, −1.8 to 1.0; P = 0.572
Baseline IL-6, pg/mL	8.8 ± 5.9	9.7 ± 7.2	—
Week-12 IL-6, pg/mL	7.6 ± 5.1	8.0 ± 5.8	—
IL-6 change from baseline to week 12, pg/mL	−1.2 ± 4.9	−1.7 ± 5.3	−0.5 pg/mL; 95% CI, −2.2 to 1.2; P = 0.561
Baseline TNF-α, pg/mL	12.1 ± 5.3	12.8 ± 5.7	—
Week-12 TNF-α, pg/mL	11.6 ± 4.9	12.1 ± 5.2	—
TNF-α change from baseline to week 12, pg/mL	−0.4 ± 3.6	−0.7 ± 3.8	−0.2 pg/mL; 95% CI, −1.3 to 0.9; P = 0.721

Values are presented as mean ± standard deviation or adjusted effect estimate with 95% confidence interval. Adjusted treatment effects were estimated using propensity score weighting with robust standard errors. Negative values for pain score, β-CTX, CRP, IL-6, and TNF-α indicate reduction from baseline. Positive values for P1NP and osteocalcin indicate increase from baseline. CI, confidence interval; P1NP, procollagen type I N-terminal propeptide; β-CTX, β-C-terminal telopeptide of type I collagen; CRP, C-reactive protein; IL-6, interleukin-6; TNF-α, tumor necrosis factor-α.

### 3.4 Radiographic healing, recurrent fracture, and BMD change

At 6 months, radiographic healing was documented in 37 of 86 patients in the standard-treatment group and 42 of 82 patients in the Xiaotong plaster adjunctive-treatment group. Delayed union or nonunion was recorded in 22 patients and 12 patients, respectively. After propensity score weighting, neither 6-month radiographic healing nor delayed union or nonunion differed significantly between groups.

During 12-month follow-up, new fragility fracture occurred in 17 patients in the standard-treatment group and 9 patients in the adjunctive-treatment group. In the weighted Cox model, Xiaotong plaster adjunctive treatment was not significantly associated with 12-month new fragility fracture risk. The proportional hazards assumption was not violated according to Schoenfeld residual testing (P = 0.742). Twelve-month BMD changes at the lumbar spine, femoral neck, and total hip were small in both groups and did not differ significantly after adjustment. Systemic anti-osteoporosis treatment persistence and all-cause readmission were also not significantly different between groups (**Table 5**; **Figure 5**).

**Table 5:** Six-month and 12-month clinical outcomes.

Outcome	Standard treatment only *n* = 86	Standard treatment plus Xiaotong plaster *n* = 82	Adjusted treatment effect
6-month radiographic healing	37/86 (43.0%)	42/82 (51.2%)	OR, 1.14; 95% CI, 0.57 to 2.29; P = 0.711
6-month delayed union or nonunion	22/86 (25.6%)	12/82 (14.6%)	OR, 0.66; 95% CI, 0.27 to 1.63; P = 0.363
12-month new fragility fracture	17/86 (19.8%)	9/82 (11.0%)	HR, 0.48; 95% CI, 0.21 to 1.10; P = 0.083
12-month MG exacerbation	9/86 (10.5%)	8/82 (9.8%)	OR, 0.79; 95% CI, 0.24 to 2.60; P = 0.692
12-month lumbar spine BMD change, %	0.23 ± 0.96	0.41 ± 1.03	0.28%; 95% CI, −0.13 to 0.69; P = 0.177
12-month femoral neck BMD change, %	0.14 ± 0.88	0.25 ± 0.91	0.18%; 95% CI, −0.19 to 0.55; P = 0.337
12-month total hip BMD change, %	0.09 ± 0.82	0.18 ± 0.87	0.14%; 95% CI, −0.22 to 0.50; P = 0.444
Documented persistence with standard anti-osteoporosis treatment ≥80%	65/86 (75.6%)	67/82 (81.7%)	OR, 1.27; 95% CI, 0.61 to 2.65; P = 0.521
All-cause readmission within 12 months	18/86 (20.9%)	15/82 (18.3%)	OR, 0.84; 95% CI, 0.38 to 1.85; P = 0.663

Values are presented as mean ± standard deviation, n/N (%), or adjusted effect estimate with 95% confidence interval. Six-month binary outcomes were analyzed using weighted logistic regression. Twelve-month new fragility fracture was analyzed using weighted Cox proportional hazards regression and reported as hazard ratio. OR, odds ratio; HR, hazard ratio; CI, confidence interval; MG, myasthenia gravis; BMD, bone mineral density.

**Figure 5:**
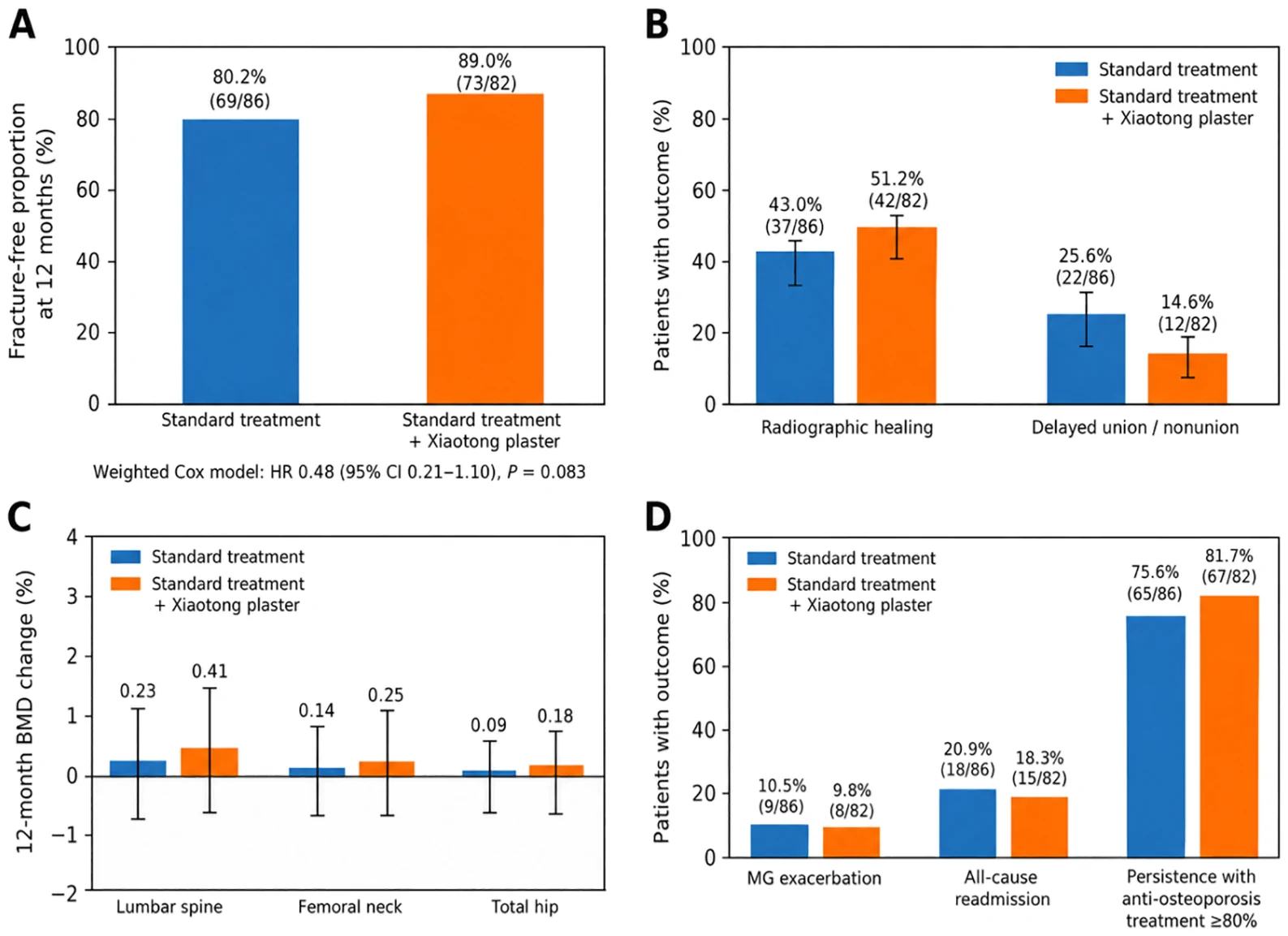
Clinical outcomes after fracture. **(A)** Bar plot of the observed 12-month new fragility fracture-free proportion by treatment group. **(B)** Bar plots with 95% confidence intervals for 6-month radiographic healing and delayed union or nonunion. **(C)** Mean 12-month BMD percentage changes at the lumbar spine, femoral neck, and total hip. **(D)** Bar plots showing 12-month MG exacerbation, all-cause readmission, and standard anti-osteoporosis treatment persistence. The 12-month fracture-free panel is descriptive and is not presented as a Kaplan–Meier estimate.

### 3.5 Exploratory peripheral-blood BMP-2/Smad/Runx2 biomarker subgroup

The exploratory BMP-2/Smad/Runx2 subgroup included 60 patients with valid paired baseline and 12-week specimens. BMP-2 increased by 8.2 ± 20.4 pg/mL in the standard-treatment group and by 14.8 ± 18.2 pg/mL in the adjunctive-treatment group, but the adjusted between-group difference was not significant. In contrast, p-Smad1/5/8 and Runx2 increased more clearly in the adjunctive-treatment group. The adjusted between-group difference was 0.142 relative units for p-Smad1/5/8 change and 0.213 relative units for Runx2 change. Because BMP-2 itself was not different between groups, the downstream marker differences were not interpreted as evidence of activation of the full BMP-2/Smad/Runx2 axis.

Within the exploratory biomarker subgroup, Runx2 change was positively correlated with P1NP change and negatively correlated with β-CTX change, while p-Smad1/5/8 change showed a weaker positive correlation with P1NP change. Runx2 change was also correlated with pain-score change (Spearman ρ = −0.278; P = 0.032), whereas BMP-2 change was not clearly correlated with the main bone-turnover changes. These analyses describe associations among exploratory peripheral markers and do not establish mediation or a local fracture-site mechanism (**Table 6**; **Figure 6**). The observed correlations cannot determine directionality and should not be interpreted as showing that biomarker changes mediated pain reduction.

**Table 6:** Exploratory peripheral-blood BMP-2/Smad/Runx2 biomarker subgroup outcomes.

Exploratory biomarker endpoint	Standard treatment only n = 26	Standard treatment plus xiaotong plaster n = 34	Adjusted between-group difference/correlation
Baseline BMP-2, pg/mL	118.4 ± 34.6	116.2 ± 31.8	—
Week-12 BMP-2, pg/mL	126.6 ± 41.2	131.0 ± 37.7	—
BMP-2 change, pg/mL	8.2 ± 20.4	14.8 ± 18.2	4.26; 95% CI, −6.77 to 15.28; P = 0.449
Baseline p-Smad1/5/8, relative units	0.83 ± 0.26	0.80 ± 0.22	—
Week-12 p-Smad1/5/8, relative units	0.85 ± 0.29	0.98 ± 0.23	—
p-Smad1/5/8 change, relative units	0.023 ± 0.180	0.178 ± 0.106	0.142; 95% CI, 0.060 to 0.224; P = 0.001
Baseline Runx2, relative units	0.91 ± 0.30	0.89 ± 0.28	—
Week-12 Runx2, relative units	0.91 ± 0.34	1.09 ± 0.31	—
Runx2 change, relative units	−0.001 ± 0.207	0.202 ± 0.204	0.213; 95% CI, 0.080 to 0.346; P = 0.002
BMP-2 change vs P1NP change	—	—	Spearman ρ = 0.184; P = 0.161
p-Smad1/5/8 change vs P1NP change	—	—	Spearman ρ = 0.291; P = 0.026
Runx2 change vs P1NP change	—	—	Spearman ρ = 0.315; P = 0.045
Runx2 change vs β-CTX change	—	—	Spearman ρ = −0.325; P = 0.033
Runx2 change vs pain-score change	—	—	Spearman ρ = −0.278; P = 0.032
Runx2 change vs 6-month healing status	—	—	Spearman ρ = 0.244; P = 0.061

Values are presented as mean ± standard deviation, adjusted between-group difference, or Spearman correlation coefficient. Analyses were restricted to patients with valid paired baseline and 12-week specimens. BMP-2, bone morphogenetic protein-2; P1NP, procollagen type I N-terminal propeptide; β-CTX, β-C-terminal telopeptide of type I collagen.

**Figure 6:**
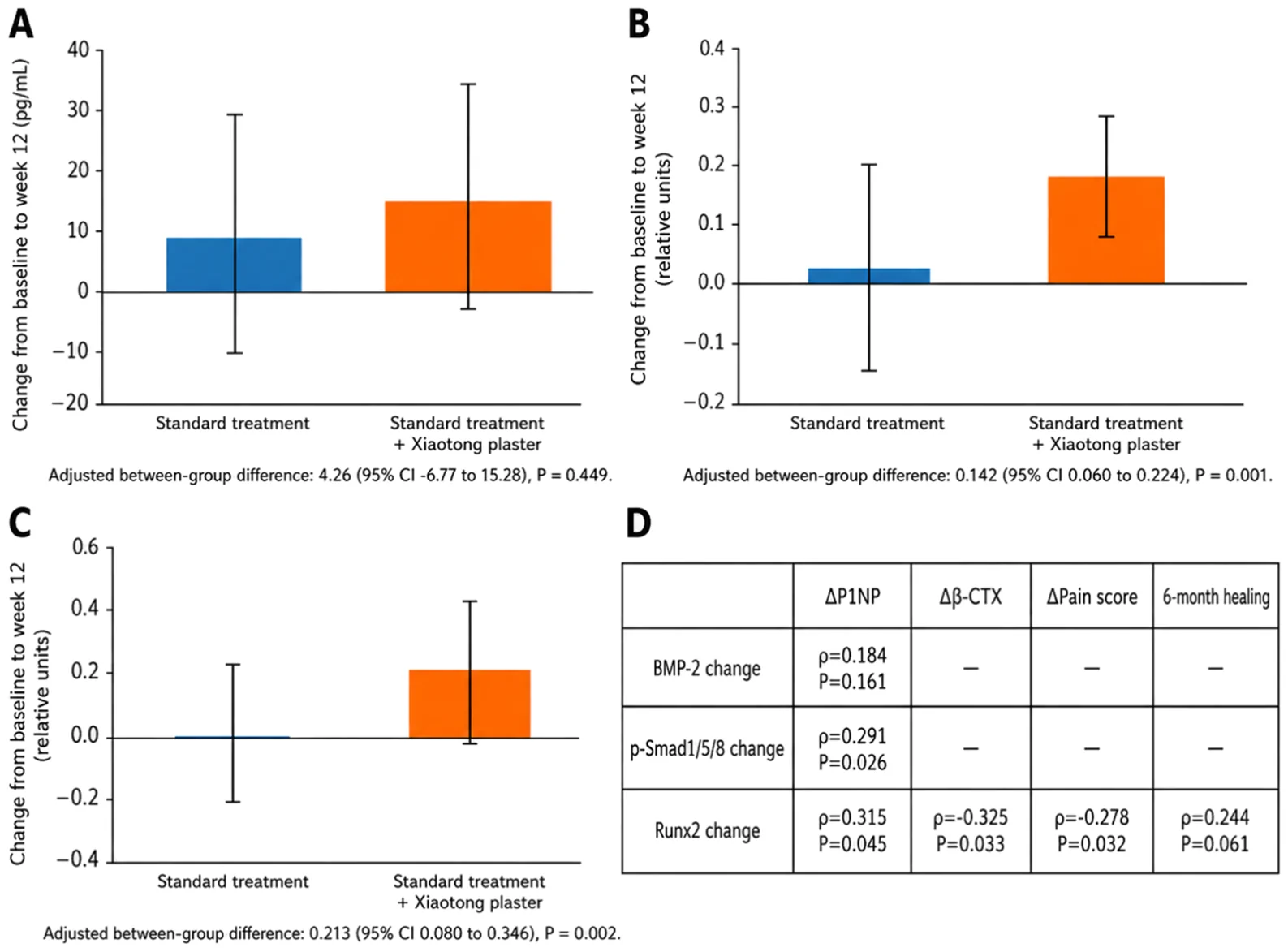
Exploratory peripheral-blood BMP-2/Smad/Runx2 biomarker analysis. **(A)** Mean changes in BMP-2 from baseline to week 12 by treatment group. **(B)** Mean changes in p-Smad1/5/8 from baseline to week 12 by treatment group. **(C)** Mean changes in Runx2 from baseline to week 12 by treatment group. **(D)** Correlation matrix showing associations between BMP-2, p-Smad1/5/8, and Runx2 changes and changes in P1NP, β-CTX, pain score, and 6-month healing status.

### 3.6 Propensity score balance, sensitivity analyses, and subgroup consistency

Before weighting, the largest baseline imbalances involved lumbar spine T-score, baseline P1NP, baseline β-CTX, teriparatide use, and vertebral compression fracture distribution. Weighting substantially improved overall covariate balance, but teriparatide use (weighted SMD, 0.128) and vertebral compression fracture distribution (weighted SMD, 0.112) remained above the target threshold of 0.10. The primary outcome was therefore additionally examined using a doubly robust weighted model retaining these residual imbalanced variables. The adjusted between-group difference remained −9.8 mm (95% CI, −13.4 to −6.1; P < 0.001).

The primary pain association remained directionally consistent in complete-case analysis, propensity score matching, stricter exposure classification, plaster-adherence restriction, standard anti-osteoporosis treatment persistence restriction, teriparatide exclusion, vertebral augmentation exclusion, and extreme-weight truncation. In the continuous exposure analysis, each additional 7 cumulative application days was associated with a further −1.7 mm change in pain score (95% CI, −2.5 to −0.9), providing an exploratory exposure-duration association. Subgroup estimates were generally consistent across age, sex, fracture site, prednisone-equivalent dose, osteoporosis severity, fracture-management strategy, and systemic anti-osteoporosis treatment type, without a clear qualitative interaction signal (**Table 7**; **Figure 2**).

**Table 7:** Sensitivity and subgroup analyses for 12-week pain reduction.

Analysis	Comparison	Estimated difference in pain change, mm	95% CI	P for interaction
Primary propensity score-weighted analysis	Xiaotong plaster vs standard treatment	−10.2	−13.8 to −6.6	—
Doubly robust weighted model	Xiaotong plaster vs standard treatment	−9.8	−13.4 to −6.1	—
Complete-case adjusted model	Xiaotong plaster vs standard treatment	−11.4	−16.0 to −6.7	—
Propensity score matching	Xiaotong plaster vs standard treatment, 63 matched pairs	−9.6	−13.9 to −5.3	—
Stricter exposure definition	≥28 cumulative application days vs standard treatment	−9.1	−12.2 to −6.1	—
Xiaotong plaster adherence restriction	≥80% documented plaster adherence vs standard treatment	−9.1	−12.6 to −5.7	—
Standard anti-osteoporosis persistence restriction	≥80% standard-treatment persistence in both groups	−9.4	−13.2 to −5.6	—
Excluding teriparatide users	Xiaotong plaster vs standard treatment	−9.0	−12.2 to −5.8	—
Excluding vertebral augmentation	Xiaotong plaster vs standard treatment	−9.7	−13.6 to −5.8	—
Extreme-weight truncation	Xiaotong plaster vs standard treatment	−10.0	−13.5 to −6.5	—
Continuous exposure analysis	Per additional 7 cumulative application days	−1.7	−2.5 to −0.9	—
Age <75 years	Xiaotong plaster vs standard treatment	−7.1	−11.2 to −3.1	0.184
Age ≥75 years	Xiaotong plaster vs standard treatment	−12.0	−16.5 to −7.4	0.184
Female patients	Xiaotong plaster vs standard treatment	−8.5	−12.0 to −5.1	0.512
Male patients	Xiaotong plaster vs standard treatment	−11.2	−17.3 to −5.0	0.512
Vertebral compression fracture	Xiaotong plaster vs standard treatment	−8.6	−13.0 to −4.2	0.773
Non-vertebral fracture	Xiaotong plaster vs standard treatment	−9.2	−13.4 to −5.0	0.773
Prednisone-equivalent dose <10 mg/day	Xiaotong plaster vs standard treatment	−11.6	−16.5 to −6.8	0.219
Prednisone-equivalent dose ≥10 mg/day	Xiaotong plaster vs standard treatment	−7.2	−10.9 to −3.4	0.219
Lumbar spine T-score >−2.5	Xiaotong plaster vs standard treatment	−8.8	−13.0 to −4.7	0.645
Lumbar spine T-score ≤−2.5	Xiaotong plaster vs standard treatment	−10.3	−15.1 to −5.4	0.645
Conservative fracture management	Xiaotong plaster vs standard treatment	−9.5	−13.4 to −5.6	0.802
Surgical fixation, arthroplasty, or vertebral augmentation	Xiaotong plaster vs standard treatment	−8.8	−14.0 to −3.7	0.802
Bisphosphonate-based therapy	Xiaotong plaster vs standard treatment	−9.3	−13.0 to −5.7	0.588
Denosumab or teriparatide-based therapy	Xiaotong plaster vs standard treatment	−8.6	−14.2 to −3.0	0.588

Negative values indicate greater pain reduction in the Xiaotong plaster adjunctive-treatment group. CI, confidence interval.

### 3.7 Safety outcomes and missing data

Plaster-related skin reaction occurred in 8 of 82 patients receiving Xiaotong plaster adjunctive treatment and in none of the patients receiving standard treatment alone. Recorded reactions included local erythema in 3 patients, pruritus in 3 patients, and contact dermatitis in 2 patients. Three patients temporarily interrupted plaster use because of skin reaction, and one discontinued plaster use permanently. No blistering requiring procedural treatment, skin ulceration, local infection, systemic allergic reaction, or hospitalization related to plaster use was recorded. All documented plaster-related reactions improved after plaster removal, local skin care, or short-term topical treatment. The permanent discontinuation due to contact dermatitis is clinically important despite the absence of serious systemic adverse events.

MG exacerbation within 12 months occurred in 9 patients in the standard-treatment group and 8 patients in the adjunctive-treatment group. Myasthenic crisis occurred in 1 patient in each group. Infection requiring antibiotic treatment occurred in 10 patients in the standard-treatment group and 9 patients in the adjunctive-treatment group. Hypercalcemia, renal-function deterioration, gastrointestinal intolerance related to systemic anti-osteoporosis treatment, and all-cause readmission were uncommon or similar between groups, without a clear safety imbalance attributable to Xiaotong plaster adjunctive treatment.

Missingness was low for demographics, MG diagnosis, MG severity, glucocorticoid exposure, fracture site, treatment exposure, baseline pain score, 12-week pain outcome, 6-month radiographic healing, 12-month new fragility fracture, and MG exacerbation. Missingness was 15.5% for week-12 P1NP and 14.9% for week-12 β-CTX. Missingness was 17.3% for week-12 osteocalcin, 18.5% for week-12 IL-6, 19.0% for week-12 TNF-α, and 16.1% for 12-month repeated BMD assessment. BMP-2/Smad/Runx2 variables were restricted to the availability-defined exploratory biomarker subgroup and were not included in the primary multivariable model. Variables with missingness of 20% or less were handled using multiple imputation by chained equations, and complete-case analysis was retained as a sensitivity analysis.

## 4 Discussion

In this retrospective cohort of elderly patients with MG, systemic glucocorticoid exposure, and osteoporotic fragility fracture, the clearest finding was an association between Xiaotong plaster adjunctive treatment and greater 12-week fracture-site pain reduction. Short-term P1NP increased more and β-CTX decreased modestly more in the adjunctive-treatment group, whereas inflammatory markers did not show a consistent between-group pattern. In contrast, 6-month radiographic healing, delayed union or nonunion, 12-month new fragility fracture, BMD change, MG exacerbation, all-cause readmission, and persistence with standard anti-osteoporosis treatment were not significantly different. The exploratory subgroup showed changes in downstream p-Smad1/5/8 and Runx2 but not BMP-2. Taken together, the evidence supports a short-term symptomatic association more strongly than immune modulation, structural skeletal benefit, or a specific osteogenic mechanism.

The study population represents a clinically narrow but important subgroup. MG-related weakness and reduced mobility can coexist with prolonged glucocorticoid exposure, and population-based studies have reported heterogeneous fracture-risk estimates in MG (4, 5). In the present cohort, baseline imbalance in lumbar spine T-score, P1NP, β-CTX, teriparatide use, and fracture-site distribution emphasized the importance of confounding control. Weighting substantially improved balance, but teriparatide use and vertebral compression fracture remained slightly above the SMD target. The consistent doubly robust and sensitivity estimates strengthen the primary association while not eliminating the possibility of residual or unmeasured confounding. In particular, the post-weighting SMDs of 0.128 for teriparatide use and 0.112 for vertebral compression fracture distribution were treated as residual imbalance rather than as evidence that complete exchangeability had been achieved.

The 12-week pain result should be interpreted separately from long-term skeletal outcomes. The adjusted difference of −10.2 mm on a 0–100 mm pain scale was statistically robust across multiple analyses, and the 12-week window corresponds to an early recovery phase in which osteoporotic fracture pain commonly changes substantially (25). However, a fracture-specific minimal clinically important difference was not prespecified for this heterogeneous fracture cohort; therefore, statistical significance should not be equated automatically with a large patient-perceived benefit. In addition, pain is a subjective outcome in an unblinded observational study and may be affected by treatment expectation, co-interventions, rehabilitation intensity, and analgesic use. The finding is best interpreted as evidence of a modest short-term symptomatic association that requires prospective controlled confirmation. Because neither patients nor clinicians were blinded and no matched placebo plaster was used, the observed pain difference cannot separate a pharmacologic effect from placebo, treatment-expectation, attention, or other contextual effects.

The bone-turnover findings provide supportive but exploratory information. P1NP and β-CTX are internationally recognized reference markers of bone formation and resorption, but they reflect systemic remodeling and are influenced by fracture status, renal function, sampling conditions, and anti-osteoporosis therapy (28). This is particularly relevant because teriparatide use remained slightly imbalanced after weighting and can directly influence bone-formation markers. The observed P1NP and β-CTX changes therefore should not be attributed solely to the plaster. They are compatible with a short-term difference in systemic remodeling, whereas the absence of a significant 12-month BMD difference argues against claiming a demonstrated structural skeletal effect.

Osteoimmunology provides a useful framework for interpreting why inflammatory and skeletal responses may diverge. Fracture healing requires a temporally coordinated inflammatory response involving immune-cell recruitment, cytokine signaling, callus formation, and later remodeling, and both excessive and insufficient inflammation can impair repair (29). In the present study, CRP, IL-6, and TNF-α did not show clear treatment-related changes. Accordingly, the data do not support a claim that Xiaotong plaster produced systemic immune modulation. The peripheral p-Smad1/5/8 and Runx2 findings may indicate pathway-associated biological variation, but BMP-2 itself was not significantly different and peripheral blood markers cannot substitute for local fracture-site tissue. These results should remain hypothesis-generating. Product-specific transdermal evidence is limited to preclinical work, including rat-skin penetration of luteolin from Xiaotong plaster (30); no human pharmacokinetic measurements in this cohort demonstrate systemic exposure sufficient to alter osteogenic signaling. A causal sequence from topical plaster to circulating BMP-2/Smad/Runx2 changes and then to fracture repair is therefore not supported.

The contrast between improved pain and unchanged longer-term outcomes is clinically informative rather than contradictory. A topical treatment may plausibly influence local symptoms without producing a sufficiently large systemic effect to alter radiographic union, BMD, or recurrent fracture risk. Moreover, glucocorticoid-associated osteoporosis is multifactorial: glucocorticoids suppress osteoblast and osteocyte function, alter remodeling, and increase skeletal fragility (19, 31), while fracture healing and BMD trajectories are also shaped by fracture type, fixation or augmentation, antiresorptive or anabolic treatment, vitamin D status, renal function, mobility, and adherence. Experimental evidence in glucocorticoid-arrested osteoblasts has also shown that BMP-related Smad activation is associated with mineralization responses (32). The study was not separately powered for each long-term endpoint, so the non-significant results should be interpreted as absence of demonstrated benefit rather than proof of no effect.

Safety findings were generally limited to local skin reactions but remain clinically relevant. Eight of 82 exposed patients developed erythema, pruritus, or contact dermatitis; three temporarily interrupted treatment and one permanently discontinued plaster use. No plaster-related ulceration, local infection, systemic allergic reaction, or hospitalization was recorded. These findings indicate that local tolerability was acceptable for most patients but that skin reactions should be discussed explicitly when considering adjunctive use in older adults. The single permanent discontinuation due to contact dermatitis should be considered a treatment-limiting adverse event and is specifically highlighted when weighing symptomatic benefit against local tolerability.

This study has several limitations. First, it was a single-center, non-randomized retrospective cohort, and propensity score weighting and doubly robust modeling cannot eliminate unmeasured confounding or treatment-selection bias. Second, the primary outcome was subjective and unblinded, making placebo or expectation effects and differences in co-interventions possible. Third, the fixed cohort size was determined by available eligible records rather than prospective power calculation; secondary, subgroup, and mechanistic analyses were not separately powered and were interpreted as exploratory without formal multiplicity correction. Fourth, medication adherence, plaster application duration, systemic analgesic exposure, and outpatient pain scores depended on routine documentation and may be misclassified. Fifth, the BMP-2/Smad/Runx2 subgroup was availability-defined and may be subject to selection bias, while peripheral biomarkers do not establish local fracture-site mechanisms. Sixth, missingness in selected laboratory and repeated BMD variables required imputation, although complete-case analysis supported the primary result. Finally, the findings require prospective, preferably blinded or placebo-controlled, multicenter confirmation before broad clinical generalization. Seventh, direct patient-level correlations between 12-week pain-score change and changes in P1NP or β-CTX were not evaluated in the final analysis, so the symptomatic and bone-turnover findings should be interpreted as parallel observations rather than as evidence that these changes tracked together within individual patients. In addition, the plaster is a multicomponent formulation, its individual active constituents were not quantified in the administered product, and no human transdermal or systemic pharmacokinetic sampling was performed; these limitations preclude attribution of the observed associations to a specific constituent or systemic molecular mechanism.

## 5 Conclusion

In elderly MG patients with glucocorticoid-associated osteoporotic fragility fracture, Xiaotong plaster adjunctive treatment was associated with greater 12-week fracture-site pain reduction than standard anti-osteoporosis treatment alone. Short-term P1NP and β-CTX changes were exploratory and, without direct patient-level correlation analyses, should not be interpreted as mechanistically coupled to pain improvement. No significant benefit was demonstrated for radiographic healing, BMD, recurrent fragility fracture, or MG outcomes. Peripheral p-Smad1/5/8 and Runx2 findings were hypothesis-generating and do not establish a causal osteogenic mechanism. Standard systemic osteoporosis treatment remains essential, and prospective controlled studies are needed to determine the clinical magnitude and reproducibility of any adjunctive symptomatic benefit. These findings support evaluation of the plaster as a symptomatic adjunct, not as a demonstrated systemic osteogenic or disease-modifying therapy.

## Data Availability

The original contributions presented in the study are included in the article/supplementary material. Further inquiries can be directed to the corresponding author.
